# A Simple Training Method to Practice Cleft Lip and Palate Markings for Trainee Surgeons

**DOI:** 10.1177/1055665620980231

**Published:** 2021-01-19

**Authors:** Rahul Padmanabhan, Shakeel Rahman, Nefer Fallico

**Affiliations:** 1 King’s College London, GKT School of Medical Education, London, UK; 2 Department of Plastic Surgery, Guy’s and St Thomas’ Hospital, London, UK; 3 The Spires Cleft Centre, Salisbury-Oxford Health NHS Foundation Trust, Salisbury, UK

Cleft centers in the United Kingdom aim to provide training to their trainees through a variety of teaching methods. Given the nature of this subspecialty and limited hands on experience, trainee surgeons can complete their respective rotations with differing levels of exposure, and ultimately, understanding compared to their peers.

Knowledge and understanding of anatomical landmarks and the geometrical aspects of cleft repair is essential. Not only for those hoping to specialize in cleft surgery but incumbent upon trainees undertaking the FRCS Plast examination in the United Kingdom. Therefore, cleft surgeons and trainers most commonly try to achieve this through real-time intraoperative practice of lip and palate markings on patients. While there is no doubt that this is beneficial to cleft fellows, plastic surgery, or max fax trainees that are offered these opportunities, this is not widely available to all trainee surgeons. Adequate exposure to cleft pathologies and opportunities to practice ought to be available to all surgical trainees taking the FRCS examination, and thus solely relying on intraoperative exposure may have some pitfalls.

First and foremost, there are not enough operations and opportunities to observe cleft surgeries around the country for all trainee surgeons to achieve adequate training. Cleft care in the United Kingdom is centralized with 10 centers offering cleft exposure to fellows and surgical trainees. At each of these centers, there will be multiple cleft fellows and surgical trainees who require the same exposure and numbers to achieve for their indicative logbook, limiting opportunities for the wider junior doctor group. Predictably, this has left many surgical trainees short of experience. A training method that reaches out to the other surgical trainees and provides them with exposure could attract more of them to cleft as a sub-speciality to develop their interest and potentially consider specializing in the field. The lack of contact some trainees currently receive has been identified by consultants as a barrier in allowing trainee surgeons to develop an interest in this field (Islam, 2019).

The COVID-19 pandemic has exacerbated this problem, requiring health care professionals to postpone elective procedures, reduce crowding in theatres, and decrease anesthetic periods for patients where possible. Couple with this, it is the demand for more efficiency and consultant led lists, making it increasingly difficult for trainee surgeons to receive intraoperative practice.

Unsurprisingly, there has been interest and research into alternative methods for cleft training, the most notable of which has been the use of simulators. With their benefits well-documented, these simulators allow trainees and surgeons to practice 3-dimensional (3D) anatomical identification and markings without the need of a patient (Schendel et al., 2005). The literature surrounding the use of simulators has yielded positive results; however, they are associated with significant costs and are not easily accessible to many hospitals (Rogers-Vizena et al., 2018). Indeed, those willing to pay for such equipment are more likely to have already committed to cleft than those who are unsure or have committed to other specialities.

**Figure 1.**
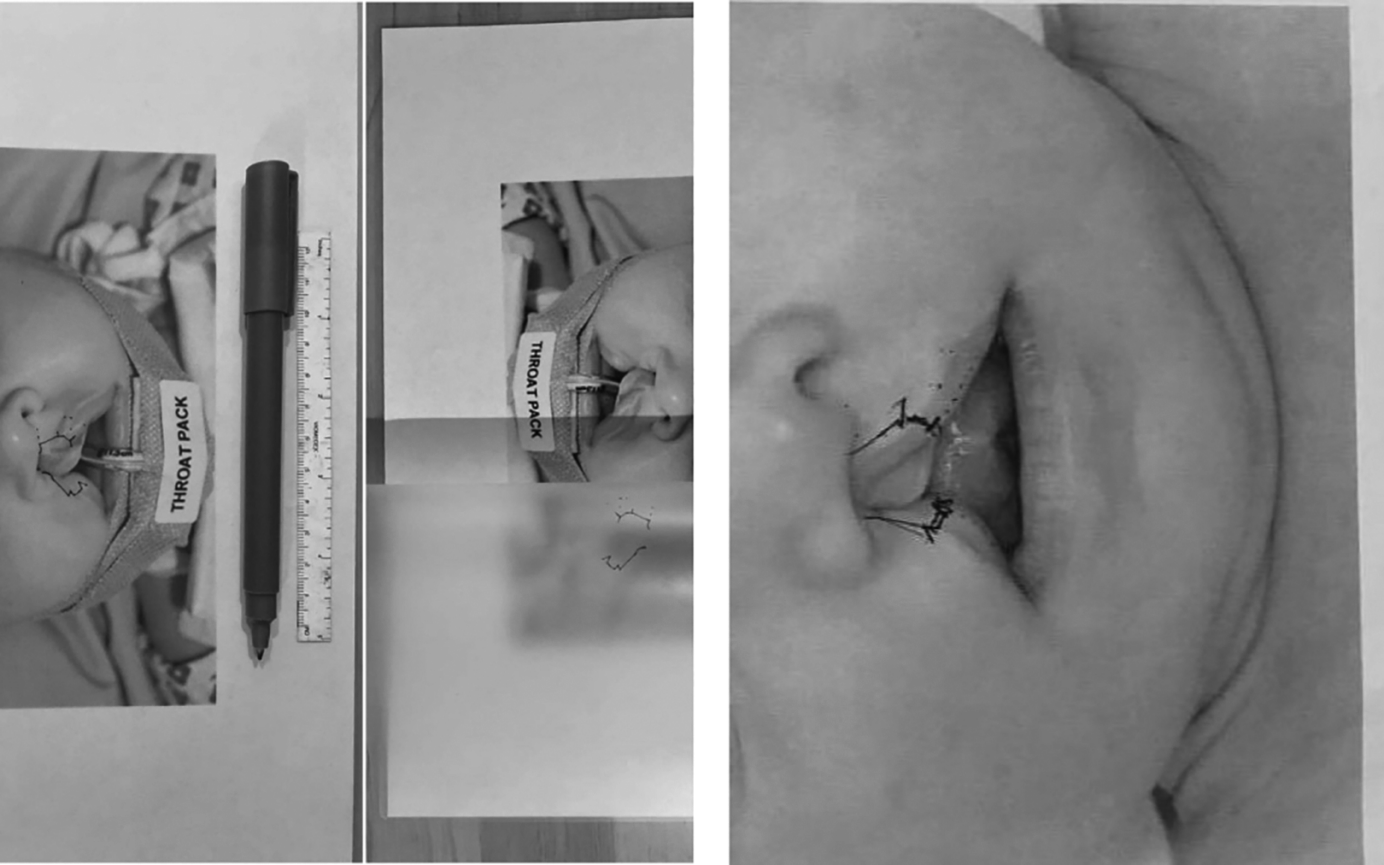
Photograph on the far left-hand side shows cleft lip and palate markings drawn on laminated pouch using non-permanent marker. Photograph immediately next to it shows cleft lip and palate markings on lifted laminated pouch. Photograph on the right-hand side shows conventional method of marking directly on photo. The markings cannot be removed and become untidy when corrected.

In an attempt to tackle these issues, we thought of a simple intervention which has been trialed at the South Thames Cleft Service and has received positive feedback. We placed clinical life size photographs of discussed cleft anomalies in a laminated pouch and asked trainees to practice their cleft lip or palate markings in fine tipped nonpermanent marker. This hands-on marking technique allows trainees to consolidate their knowledge and visualize the geometry of the repair, while enabling trainers to easily check and correct multiple attempts at any given time. The same pictures could be used repeatedly for one to one teaching or multiple trainees in a small group teaching session, without the need to organize a dedicated intraoperative training session, providing flexibility in training opportunities. Furthermore, it is a cost-effective solution that could be accessed by any hospital or doctor without difficulty. It also accurately replicates some of the conditions of real time cleft surgery compared to using an electronic mobile device, in terms of the same or similar marker pen used, accurate measurements and dimensions, and time taken to perform markings (Figure 1).

While this doesn’t replace the intraoperative experience, it does provide a cheap and easy medium through which to practice cleft lip and palate markings and yielded positive feedback. Although the lack of 3D visualization available in this method may appear disadvantageous, it did not seem to hinder the trainees but moreover seemed to replicate what is expected in the FRCS exam or FRCS preparation practice sessions. This mirrors the results of a previous study which concluded that 2-dimensional visualization of cleft repair has an acceptable reliability not differing significantly to that of 3D (Jones et al., 2018). It has also been found that active recall and the repetition of drawings for cleft lip and palate repair contributes to the progression of the learning curve toward competence, which explains the benefits of this training method (Segna et al., 2018).

Through the positive feedback received, we believe that this is a simple yet effective method to practice cleft lip and palate markings, which could benefit trainees sitting the FRCS examination and looking for exposure to cleft surgery.
